# Dynamic neural processing of self-other synchronization error in interpersonal coordination

**DOI:** 10.1016/j.isci.2025.114081

**Published:** 2025-11-17

**Authors:** Manuel Varlet, Sylvie Nozaradan, Peter E. Keller

**Affiliations:** 1 The MARCS Institute for Brain, Behaviour and Development, Western Sydney University, Penrith, NSW, Australia; 2 School of Psychology, Western Sydney University, Penrith, NSW, Australia; 3 Institute of Neuroscience (IONS), Université catholique de Louvain (UCL), Brussels, Belgium; 4 Center for Music in the Brain, Department of Clinical Medicine, Aarhus University & The Royal Academy of Music Aarhus/Aalborg, Aarhus, Denmark

**Keywords:** behavioral neuroscience, cognitive neuroscience

## Abstract

The human capacity to produce precisely synchronized actions with others is critical for everyday cooperative activities. Such interpersonal coordination is supported by dynamic neural processes that minimize discrepancies between the movements of self and others. Here we use EEG and movement data from 28 pairs of participants performing an improvised joint synchronization task to investigate how the brain continuously tracks self-other synchronization error. Results revealed a central role of the dorsal visual stream dynamically balancing the neural tracking of self-other error depending on leadership roles. We found stronger neural tracking when following, evidenced by higher EEG-error coherence, suggesting a greater need for adaptation in this role. In contrast, leading was characterized by weaker coherence that peaked earlier, suggesting greater involvement of predictive top-down processes driven by motor regions, facilitated by knowledge of upcoming self-other error. Our findings provide insights into key neural processes underpinning interpersonal coordination and related disorders.

## Highlights

• The human brain selectively tracks self-other synchronization error in joint action
• The neural tracking is leadership-dependent, supported by the dorsal visual stream
• Following role shows stronger neural tracking, indicating greater adaptation needs
• Leading role shows shorter latency neural tracking, suggesting motor-based prediction

## Graphical abstract

**Figure:**
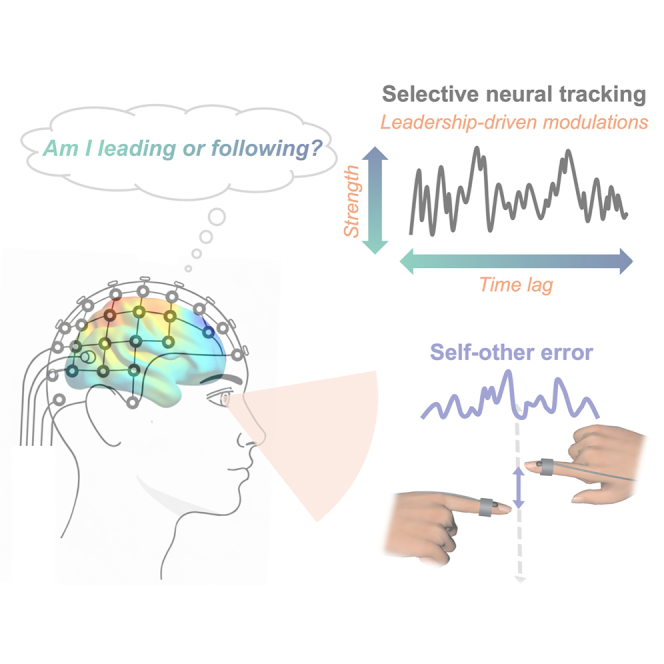

## Introduction

Establishing and maintaining precisely synchronized actions with others, including humans as well as artificial agents, is critical in a wide range of cooperative activities. Synchronization errors of few tens of milliseconds can considerably impact collective performance and even safety, such as in team sports like rowing or soccer, military squad movements, human-machine interaction, or musical ensembles.^1^^,^^2^^,^^3^^,^^4^^,^^5^ Synchronized movements between agents also act as social glue in everyday life, increasing interpersonal bonding, connectedness, affiliation, and pro-social behaviors.^6^^,^^7^^,^^8^^,^^9^ Larger interactional synchrony errors have been linked to social disorders occurring in mental illnesses such as schizophrenia, in autism spectrum disorder, and with social phobia, and therefore, are emerging as potential biomarker for earlier diagnoses and interventions.^10^^,^^11^^,^^12^^,^^13^^,^^14^

Self-other synchronization errors are unlike other errors faced by the human brain. They are the product of mutual adaptations of two or more complex biological systems underpinned by dynamic bidirectional couplings.^15^^,^^16^^,^^17^ Due to constant changes in the dynamics of the multiple components—self, other, and their coupling—monitoring and predicting self-other errors is computationally challenging, especially during improvised interpersonal synchronization performance in which actions are not pre-planned.^16^^,^^18^^,^^19^^,^^20^ While the brain mechanisms supporting the processing of self-other error are not fully understood, it has been proposed that they might be facilitated by conditions easing error prediction through motor-based top-down predictive processes.^18^^,^^19^^,^^21^^,^^22^^,^^23^^,^^24^ Previous studies with neuroimaging and brain stimulation techniques have provided evidence for motor-based predictions in musical settings related to when and what a partner is playing.^22^^,^^23^^,^^25^^,^^26^ However, whether similar processes support visually based interpersonal movement synchronization performance, in which individuals have access to continuous movement and synchronization information error, has not been established.

Moreover, whether or not leadership roles are assigned is a decisive factor in improvised interpersonal synchronization performance, strongly modulating individual and collective behavioral dynamics.^18^^,^^27^^,^^28^^,^^29^ Visually guided interpersonal synchronization in improvised scenarios has been found to improve, as reflected by smaller self-other synchronization errors, when there is no designated leader.^28^^,^^29^ Designated leader and follower roles lead to asymmetric coupling, with the follower lagging behind the leader, and the leader and follower being more focused on self or other, respectively, as observed by^29^ using EEG frequency tagging to overcome motor-related artifacts and track the neural processing of these two sources of information. In contrast, when no leader was designated, this research revealed a more balanced processing of self and other with enhanced neural integration of these two sources of information, which might constitute a prerequisite for optimal processing of self-other errors at an early stage of the visual pathway.

**Figure 1:**
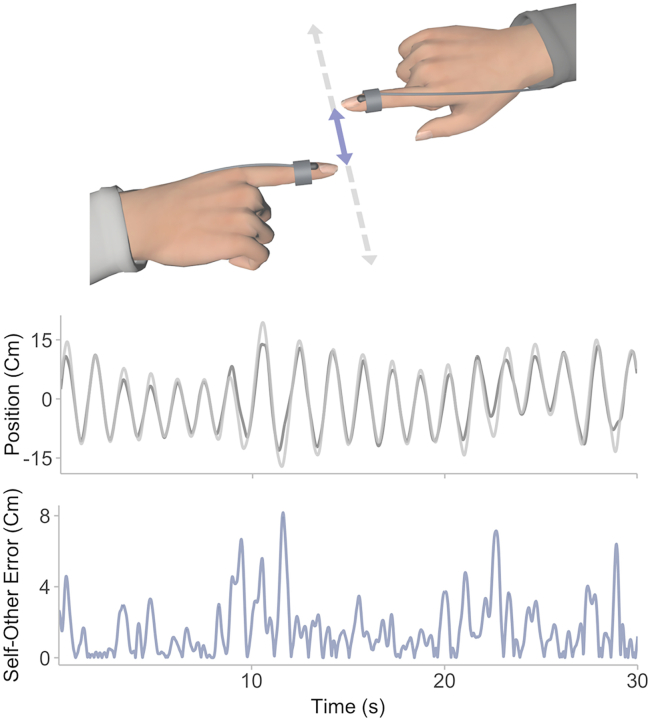
Illustration of the interpersonal improvisation synchronization task Time series represent 30 s of representative movement data from two participants (light and dark lines in top plot) with the corresponding self-other error (bottom plot).

Here we investigated how variations in demands and predictability induced by different leadership roles modulate the neural processing of visual self-other synchronization errors. We took advantage of the dual-EEG dataset of,^29^ where 28 pairs of participants performed an improvised interpersonal synchronization task, to undertake a novel examination of the processing of dynamic and continuous self-other errors in the visual domain. The task was a version of the mirror game in which participants had to produce original and synchronized continuous horizontal forearm oscillations with and without designated leadership roles,^28^ as illustrated in Figure 1. We used motion-capture data to measure instantaneous self-other synchronization error together with EEG recordings to examine how this error is processed by the brain as a function of an individual’s leadership role (i.e., follower, leader, and joint) using EEG-error cross-spectral coherence, enabling us to measure the neural tracking of dynamic multifrequency inputs.^30^^,^^31^ Using source modeling of EEG signals, we hypothesized that the dorsal visual stream would play a central role in the processing of self-other error, as reflected by higher coherence in related areas, due to its key function in perception for action.^32^^,^^33^^,^^34^ We also expected leadership roles to modulate the magnitude and temporal dynamics of self-other error processing along the dorsal visual stream.

Specifically, we predicted stronger EEG-error cross-spectral coherence when following due to the greater need for adaptation and predicted earlier peaks in EEG-error coherence when leading, which could reflect stronger involvement of top-down processes along the dorsal visual stream due to participants’ greater capacity to anticipate potential discrepancies between their own movements and those of their partner (self-other errors). While improvising, leaders arguably have more precise knowledge about the spatiotemporal movement variations and related self-other errors that might occur next as they initiate, and therefore they can better predict, upcoming motor and coordination patterns.^19^^,^^27^^,^^28^ In other words, leaders can anticipate changes in movement direction, frequency, and/or amplitude seconds in advance of initiating them while trying to achieve new, original movement dynamics, whereas followers are often limited to reacting to these changes during improvisation. This knowledge was expected to enable leaders to make sensory predictions about fluctuations in self-other errors accordingly.^16^^,^^19^^,^^21^^,^^24^^,^^35^ Such predictive processing enabled by prior knowledge when leading was expected to be reflected in earlier peaks in EEG-error coherence, particularly in motor-related regions, compared to when following.

## Results

EEG and motion-capture recordings together revealed that the brains of participants jointly improvising cooperative synchronized actions directly track the visual error between self- and other-generated movements and that this neural tracking is selectively modulated by leadership roles—follower, leader, and joint leadership—influence (i) the magnitude and frequency range of self-other error tracking and (ii) the underlying spatiotemporal brain dynamics of self-other error tracking.

### Magnitude and frequency range of visual self-other error tracking

**Figure 2:**
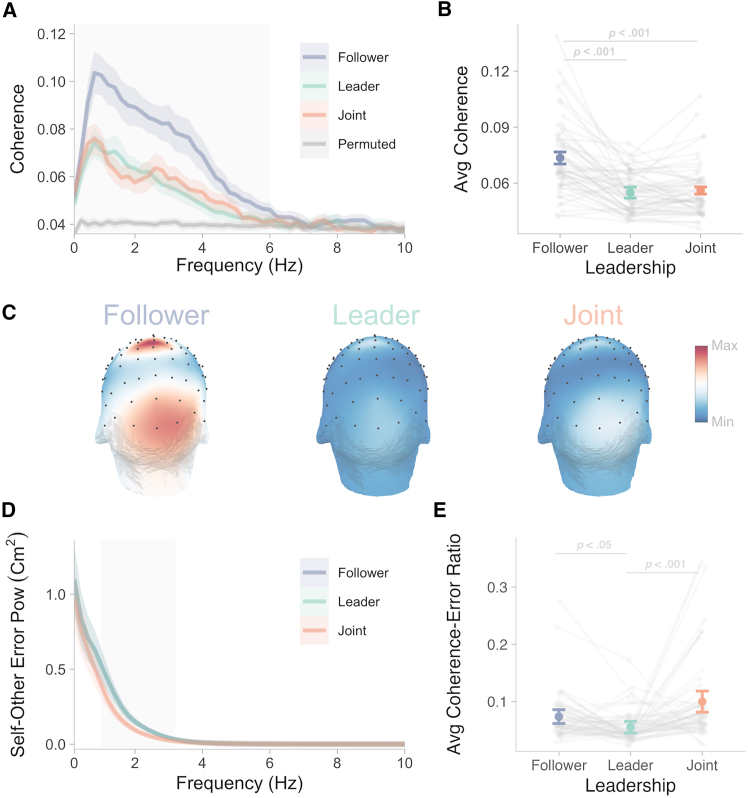
Plots of coherence between EEG and self-other error, and self-other error magnitude, across leadership roles EEG-error coherence is shown for the different leadership conditions averaged across all electrodes for the different frequencies in panel (A) and averaged within the significant 0–6 Hz frequency range in panel (B) with the corresponding topographical maps in panel (C). Panel (D) represents the magnitude (power) of self-other error and panel (E) represents EEG-error coherence normalized by self-other error magnitude (power) for each frequency and averaged within 0–6 Hz (i.e., Avg coherence-error Ratio). Gray shaded areas indicate clusters of coherence values significantly higher in real than permuted data (i.e., 0–6 Hz) in panel (B), and of self-other error magnitude (power) significantly lower in Joint than in follower and leader in panel (E). Colored shaded areas and error bars represent 1 × 95% CI of the mean computed for within-subject designs.^36^

Participants’ EEG activity was synchronized with continuous changes in self-other error up to 6 Hz approximately, as indicated by cluster-based permutation testing yielding significantly higher coherence values (averaged across all EEG electrodes and leadership roles) in real than permuted data (see Figure 2A). This difference between real and permuted data show that coherence up to 6 Hz was significantly above chance, not trivially driven by the power spectrum of self-other error, but rather reflecting genuine neural tracking. The range of frequencies tracked is generally in line with the frequency range of errors showing larger amplitudes—slower frequency errors with larger amplitudes tended to be associated with higher coherence—as reflected by a significant positive correlation between power and coherence values across frequencies (M = 0.39, 95% CI ± 0.046, *t*[55] = 16.88, *p* < 0.0001, *d* = 2.26, BF_10_ > 100), and as seen when comparing panels A and D of Figure 2. However, the magnitude of the errors did not fully explain the magnitude of the coherence, as suggested by this only moderate positive correlation. Most importantly, relatively high EEG-error coherence was observed at higher frequencies (beyond 2–3 Hz; see Figure 2A) despite much lower error amplitudes in that range (see Figure 2D). This suggests that the processing of fast and low amplitude errors could be centrally enhanced to enable finely tuned behavioral synchronization.

Moreover, a one-way repeated-measures ANOVA with the three level factor leadership on EEG-error coherence values averaged within this frequency range revealed a significant effect, *F*(2, 110) = 57.83, *p* < 0.0001, *η*_*g*_^*2*^ = 0.24, BF_10_ > 100 (see Figure 2B). Pairwise comparisons (three tests in total, with Bonferroni correction) yielded stronger neural tracking of visual self-other error when a participant was instructed to follow compared to lead, *t*(55) = 7.77, *p* < 0.0001, *d* = 1.18, BF_10_ > 100, or to do the task jointly without designated leader, *t*(55) = 9.73, *p* < 0.0001, *d* = 1.02, BF_10_ > 100. Topographical maps in Figure 2C indicate responses widely distributed under parieto-occipital electrodes consistent with selective tracking of self-other error in visual regions, as confirmed below with source analyses.

Nevertheless, participants synchronized their movement better when doing the task jointly without a designated leader, in line with previous research, resulting in lower error (power) magnitude as seen in Figure 2D. When normalized by the power of the error for each frequency in the 0–6 Hz range, average EEG-error coherence values were found to be larger without a designated leader, *F*(2, 110) = 10.30, *p* < 0.001, *η*_*g*_^*2*^ = 0.12, BF_10_ > 100, as depicted in Figure 2E. Pairwise comparisons (3 tests in total, with Bonferroni correction) yielded greater values for joint compared to leader, *t*(55) = 4.03, *p* < 0.001, *d* = 0.81, BF_10_ > 100, suggesting stronger neural tracking in this condition when taking into account the difference in the magnitude of the visual error.

**Figure 3:**
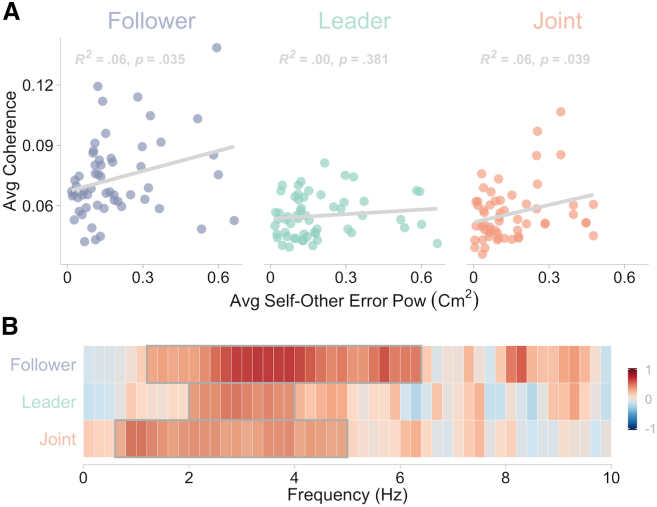
Correlation between EEG-error coherence and self-other error magnitude Panel (A) shows the correlation averaged within the significant 0–6 Hz frequency range and panel (B) depicts the correlation for each frequency. The diagonals in panel (A) represent the line of best fit with the corresponding R-squared and *p* values. Gray rectangles in panel (B) indicate significant clusters of correlation revealed by permutation testing.

The link between the magnitude of EEG-error coherence and self-other error was also further evidenced by significant correlations between the two measures across participants, especially for follower and joint. Specifically, larger self-other error was associated with greater EEG-error coherence. Significant positive correlations were found between the average coherence and the magnitude (power) of self-other error averaged in the 0–6 Hz range only for follower and joint, as depicted in Figure 3A. Analyses conducted across all frequencies indicated that EEG-error coherence correlated with the magnitude of the error also for leader but for a reduced range of frequencies compared to follower and joint (see Figure 3B). Notably, no significant correlation was found for the slowest frequencies, despite self-other error being larger in magnitude. The strongest correlations were generally observed above 2 Hz, where self-other errors had much lower magnitudes (see Figure 2D). This further emphasizes that low-amplitude, faster frequency errors might be more critically processed and centrally enhanced compared to large-amplitude, slower frequency errors.

### Spatiotemporal brain dynamics of self-other error tracking

**Figure 4:**
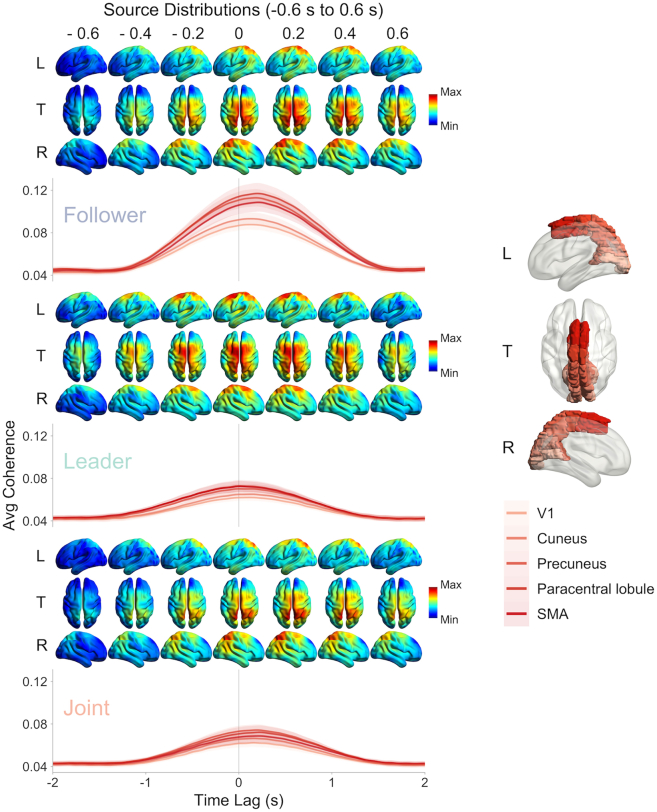
EEG-error coherence in source space across time lags and leadership roles Grand averaged time course of EEG-error coherence is depicted as a function of the different leadership roles (Follower, Leader, Joint) and time lags (from −-2 s to 2 s) for five regions of interest from early visual cortex to motor regions through the dorsal visual stream (V1, cuneus, precuneus, paracentral lobule, and SMA, parcels from the AAL [Automated Anatomical Labeling] atlas^38^) with the corresponding source distributions (from −0.6 s to 0.6 s). Source distributions are shown for a truncated time window of −0.6 s–0.6 s, subset of the full −2 s–2 s interval. Positive time lag values indicate brain modulations lagging behind self-other error and negative values indicate brain modulations occurring ahead of self-other error. Colored shaded areas represent 1 × 95% CI of the mean computed for within-subject designs.^36^

EEG-error coherence analyses were also conducted in source space using beamformer techniques^37^ to enable finer examination of the brain regions underlying the processing of self-other error. These analyses were performed in the 0–6 Hz range, in line with the significant range of frequencies observed across the whole brain in the channel space analyses, and over varying error time lags from −2 s to 2 s to examine the temporal dynamics of the processing of self-other error across brain regions and leadership roles. These analyses showed responses along the dorsal visual stream, compatible with visual processing for action, that were modulated by leadership role. Figure 4 depicts EEG-error coherence values for five regions of interest (five parcels from the AAL [Automated Anatomical Labeling] atlas^38^)—V1, cuneus, precuneus, paracentral lobule, and SMA—from early visual cortex to motor regions through the dorsal visual stream for the different self-other error time lags and leadership roles (i.e., follower, leader, and joint) with the corresponding averaged source distributions. The results revealed that not only the magnitude of EEG-error coherence in these regions varied as a function of participants’ leadership role, as shown in the channel space, but also the time at which coherence peaked in these regions, in line with knowledge-based predictive processing.

A two-way ANOVA on the maximum coherence magnitude with the factors leadership and region indicated a significant main effect of leadership, *F*(2, 110) = 68.23, *p* < 0.0001, *η*_*g*_^*2*^ = 0.25, BF_10_ > 100. Maximum EEG-error coherence was higher when following (M = 0.10, 95% CI ± 0.004) compared to when leading (M = 0.07, 95% CI ± 0.003), *t*(55) = 8.41, *p* < 0.0001, *d* = 1.15, BF_10_ > 100, and when doing the task jointly without designated leader (M = 0.07, 95% CI ± 0.002), *t*(55) = 10.68, *p* < 0.0001, *d* = 1.07, BF_10_ > 100, in line with channel space analyses. The ANOVA also revealed a significant effect of region, *F*(4, 220) = 30.69, *p* < 0.0001, *η*_*g*_^*2*^ = 0.05, BF_10_ > 100. Maximum EEG-error coherence was the lowest in V1 (M = 0.072, 95% CI ± 0.003, lower than all other regions, all *p* values <0.0001) and then cuneus (M = 0.076, 95% CI ± 0.003, lower than precuneus, paracentral lobule, and SMA, all *p* values <0.005). Coherence in precuneus (M = 0.087, 95% CI ± 0.005), paracentral lobule (M = 0.089, 95% CI ± 0.005), and SMA (M = 0.085, 95% CI ± 0.004) did not differ between precuneus and paracentral lobule and between precuneus and SMA (all *p* values >0.05), but was significantly lower in SMA compared to paracentral lobule, *t*(55) = 3.48, *p* = 0.01, *d* = 0.19, BF_10_ = 0.19. Note that caution is warranted concerning this significant difference between SMA and the paracentral lobule, given that the Bayes factor is below 1. The ANOVA also indicated a significant interaction between the two factors, *F*(8, 440) = 16.19, *p* < 0.0001, *η*_*g*_^*2*^ = 0.02, BF_10_ > 100. Pairwise comparisons revealed that EEG-error coherence was higher in the follower condition compared to the leader and joint conditions in all regions of interest (all *p* values <0.0001), and that significantly lower EEG-error coherence in SMA compared to the paracentral lobule was only found when following, *t*(55) = 4.48, *p* = 0.001, *d* = 0.20, BF_10_ = 0.33 (*p* values >0.05 in other conditions), suggesting a lower relative contribution of SMA in this leadership condition. This significant difference between SMA and the paracentral lobule also warrants caution, given that the Bayes factor is below one.

A two-way ANOVA on the time lags at which EEG-error coherence reached it maximum magnitude with the factors leadership and region indicated a significant main effect of leadership, *F*(2, 110) = 6.85, *p* = 0.002, *η*_*g*_^*2*^ = 0.05, BF_10_ > 100. Although all time lags were generally positive, reflecting delayed processing of self-other error, pairwise comparisons (three tests, with Bonferroni correction) indicated smaller maximum coherence time lags (closer to 0 s) when leading (M = 0.04, 95% CI ± 0.02) compared to when following (M = 0.10, 95% CI ± 0.01), *t*(55) = 3.40, *p* = 0.004, *d* = 0.51, BF_10_ > 100, and when doing the task jointly without designated leader (M = 0.10, 95% CI ± 0.02), *t*(55) = 2.85, *p* = 0.02, *d* = 0.40, BF_10_ > 100, showing earlier processing of visual self-other error. These results align with the occurrence of predictive processing based on prior knowledge of upcoming self-other error when leading.

The interaction between Leadership and Region did not reach statistical significance but the Bayes factor indicated strong evidence for the alternative hypothesis, *F*(8, 440) = 1.62, *p* = 0.12 *η*_*g*_^*2*^ = 0.009, BF_10_ > 40.1. As Bayesian statistics provide a powerful alternative to frequentist statistics,^39^^,^^40^ pairwise comparisons (15 tests in total) with Bonferroni correction across conditions for each brain region were conducted to examine the nature of this interaction. Although these results need to be interpreted with caution due to the non-significant result obtained with the frequentist approach, they suggest that while there was no difference in the timing of maximum coherence between follower and leader for V1 and the cuneus region of interest, maximum coherence when following occurred later (more positive lags) than when leading for the precuneus, *t*(55) = 3.96, *p* = 0.003, *d* = 0.79, BF_10_ > 100, paracentral lobule, *t*(55) = 3.77, *p* = 0.006, *d* = 0.73, BF_10_ > 100, and SMA, *t*(55) = 3.30, *p* = 0.026, *d* = 0.62, BF_10_ = 23. These differences were also supported by one-way ANOVAs conducted on each leadership role separately, which indicated a significant effect of region only when following, *F*(4, 220) = 5.69, *p* = 0.005, *η*_*g*_^*2*^ = 0.04, BF_10_ = 1.54. These results are consistent with the occurrence of motor-driven predictive processing of self-other error when leading.

These results suggest that visual information related to self-other error reaches later processing stages along the dorsal stream and then motor regions, when following. This delay does not seem to be observed when following despite generally higher coherence magnitude, potentially consistent with the involvement of stronger bottom-up processes and reduced anticipation. A corresponding delay was not observed when leading and largely decreased when doing the task jointly without a designated leader, suggesting stronger involvement of top-down processes implicating motor regions, which could be due to greater capacity to predict upcoming changes in movements and self-other error.

**Figure 5:**
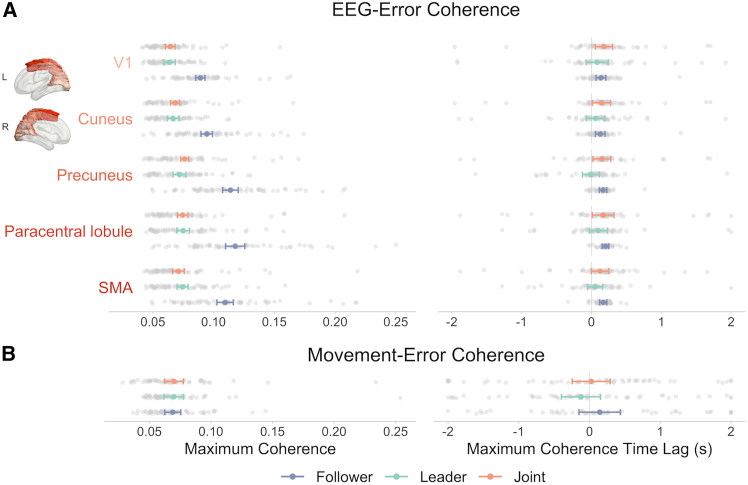
Maximum coherence with self-other error and the corresponding time lag across leadership roles Panel (A) depicts the maximum coherence (left panel) and its corresponding time lag (right panel) as a function of the different leadership roles (follower, leader, and joint) for the five regions of interest (V1, cuneus, precuneus, paracentral lobule, and SMA, parcels from the AAL [Automated Anatomical Labeling] atlas^38^). Panel (B) shows the maximum coherence (left panel) and its corresponding time lag (right panel) for participants’ actual movement (changes in instantaneous frequency). Positive time lag values indicate EEG or movement modulations lagging behind self-other error and negative values indicate EEG or movement modulations occurring ahead of self-other error. Error bars represent 1 × 95% CI of the mean computed for within-subject designs.^36^

Coherence between self-other error and participants’ actual movements (changes in instantaneous frequency) was found to be generally low with no significant differences in maximum coherence time lags across leadership roles, *F*(2, 110) = 0.90, *p* = 0.41, *η*_*g*_^*2*^ = 0.01, BF_10_ = 0.13 (see Figure 5B). This suggests that time differences in EEG-error coherence across conditions were unlikely to be driven by the degree to which movements were aligned with self-other error. Significant differences similar to those observed for EEG would have been expected if movements were driving the effect. Instantaneous frequency enabled controlling for changes in movement alignment with self-other error that could have contributed to EEG-error coherence. It reflects slight adjustments (increases and decreases) in a participant’s movement frequency and varies (similarly to self-other error) largely independently from forearm and eye movements (exhibiting continuous sinusoidal modulations; see supplemental information for further details). Moreover, the symmetric distribution of EEG-error coherence across the two hemispheres provides converging evidence that the observed effects are unlikely to be due to differences in movement synchronization to the extent that EEG-error coherence linked directly to movement would have been largely distributed over the left hemisphere, as participants were moving their right arm. Finally, additional control analyses presented in the supplemental information suggest that participants’ eye movements had a minimal contribution to the neural tracking of self-other error and associated effects. Participants continuously tracked the produced movements with their eyes, as reflected by high levels of coherence between electrooculography data and participants’ forearm movements (supplemental information Figure S3). Accordingly, high levels of coherence were also observed between EEG and participants’ movements due to muscular activity from eye movements propagating to EEG, as reflected by maximum coherence distributed laterally over frontal channels near the eyes (supplemental information Figure S4). In contrast, much lower coherence levels were observed between EEG and self-other error, with maximum coherence distributed over more posterior channels, far from the eyes, suggesting distinct activities and processes.

## Discussion

Our study provides evidence that self-other synchronization error, a wide range of human activities requires to be minimized to ensure optimal collective performance and successful social interaction, is continuously tracked by the human brain. We show that the neural tracking of such continuous error is achieved through dynamic leadership-dependent selective processes along the dorsal visual stream. More generally, this novel hyperscanning approach holds promise to provide fresh insights into the neural processes supporting collective performance and communication.^1^^,^^16^^,^^28^^,^^41^^,^^42^

EEG recordings revealed neural activity in the visual regions of the brain synchronized with continuous changes in self-other synchronization error, showing that relevant information for effective real-time coordination with others is already available at early processing stages. This neural tracking of visual self-other error was observed across a wide range of frequencies, from slow and large amplitude errors to fast (at least 6 Hz) and small amplitude errors, therefore providing information required to optimize movement and synchronization across multiple time scales relevant to natural social interaction.^15^^,^^17^^,^^43^^,^^44^^,^^45^ Furthermore, analyses in source space suggested that neural activity synchronized to self-other error was largely distributed in brain regions along the dorsal visual stream, which is known to provide rapid and flexible processing of visual information for the control of human actions.^32^^,^^34^ Our findings provide evidence that the role played by the dorsal visual stream is not only critical for the control of individual actions such as grasping or pointing but also for the control of cooperative actions by supporting the processing of visual self-other synchronization error.^33^^,^^46^

Remarkably, EEG results also revealed that the strength of the tracking and the timing of the information processing of visual self-other error along the dorsal visual stream depend on an individual’s leadership role. The results show that the tracking of self-other error is stronger when following than leading, as indicated by higher alignment of neural activity with self-other error and for a wider range of frequencies. These findings likely reflect an individual’s greater effort and processing load to maintain synchronization when following in line with previous behavioral research that showed asymmetric behavioral coupling with followers adapting more to the leaders than the inverse.^27^^,^^28^^,^^29^ However, while the tracking of visual self-other error is stronger when following, our results also suggest that it takes longer for the brain to process visual information related to self-other error when following, as indicated by delayed maximum EEG-error coherence compared to when leading. Furthermore, the results also revealed a delay between maximum coherence in motor regions compared to earlier visual regions when following, which might reflect the time required for self-other information to be processed and travel along the dorsal visual stream and reach motor regions.

Such differences were not observed when leading, suggesting that maximum EEG-error coherence occurred simultaneously in all regions—from early visual cortex to motor regions through the dorsal visual stream—and earlier than when following. These results support the involvement of top-down processes when leading, underpinned by predictive mechanisms driven from motor regions, due to the leader’s greater capacity to predict incoming self-other error. They are in line with growing evidence of predictive sensory processing observed in a variety of tasks with human neuroimaging, in which motor regions have been found to play a central role through action simulation.^21^^,^^24^^,^^35^ Here we suggest that such processes are also critical for interpersonal synchronization in improvised performance, especially when an individual leads in contrast to when an individual follows, which appears, as seen in EEG results, largely dependent on bottom-up processes due to lower capacity to predict incoming self-other error.

In the joint condition without leader-follower instructions, our EEG results suggest an advantage of undesignated leadership in enabling optimal balance between predicting and adapting to self-other synchronization error. The intensity and timing of the processing of visual self-other error when doing the task jointly without designated leader were generally intermediate to those observed in the leader and follower conditions. Strong tracking despite reduced self-other error amplitude, extending to a wider range of frequencies with reduced information processing delay, might enable more effective synchronization observed in the joint condition. More generally it can be argued that similarity between two individuals’ self-other processing when synchronizing jointly without designated leader supports synchronization whereas dissimilarity when one is leading and the other one is following is detrimental to synchronization. Future research could further explore how similarity in self-other processes is achieved in such joint action dynamics—whether by maintaining a constant symmetric relationship or by alternating between leader and follower roles.

Our findings extend previous research on self-other neural processes, providing first evidence of dynamic processes in visuo-motor brain regions supporting the processing of self-other movement synchronization error. The spatial distribution of these processes contrasts with those previously revealed in more fronto-parietal brain regions.^23^^,^^47^^,^^48^^,^^49^^,^^50^^,^^51^^,^^52^^,^^53^ We argue that these processes are complementary and that those revealed here, potentially more directly supported by sensory and motor regions, might reflect lower-level neural processes that have developed to enable rapid and continuous low-level movement adjustments required for finely tuned synchronization behavior. Responses in fronto-parietal regions observed in previous research might reflect higher-level mechanisms supporting more abstract encoding of self-other errors inherent to the synchronization of discrete actions such as during joint music making.^16^^,^^23^^,^^50^^,^^53^ Our findings encourage future research exploring the complementarity of these processes and their optimal balance for effective and flexible self-other error processing.

Beyond these findings, the current methods open new avenues for future research aimed at better understanding the brain mechanisms underpinning self-other synchronization and collective performance in everyday cooperative tasks.^15^^,^^16^^,^^20^ The combination of EEG and motion capture methods, together with cross-spectral coherence analyses to track self-other error, enabled robust measurement of the neural processing of dynamic and natural self-other error to be obtained despite potential interference from participants continuously moving. These methods extend previous work on this dataset focused on the tracking and integration of self and other movement information^29^ by revealing self-other error information in the visual system directly relevant for fine control of interpersonal coordination. While frequency tagging was initially critical to track self- and other-related information in these ecological settings due to contamination of the EEG by eye and forearm movement-related activity, here we show that this issue is less relevant when examining self-other error neural processing. Indeed, self-other error variance appeared here largely independent from that of eye and forearm movements, enabling its neural tracking to be captured straightforwardly and meaningfully with coherence measures (see supplemental information for further details).

By providing objective measures of the processing of real-time self-other synchronization error, these methods are especially relevant to examining interindividual differences beyond the influence of leadership roles investigated in this study. Future research could assess post-improvisation psychological factors such as feelings of connectedness and togetherness experienced by participants using interviews to examine their relationship with self-other error processes.^8^^,^^54^ Further, mental disorders and expertise have been shown to influence interpersonal synchronization in previous behavioral research.^1^^,^^10^^,^^11^^,^^13^^,^^14^ However, the nature of the control processes affected remains unclear. The methods developed here make it possible to investigate these modifications at a neurophysiological level in natural settings, which could ultimately help in developing more effective diagnosis and interventions for patients suffering from schizophrenia, autism spectrum disorder, and social phobia, for instance.

More generally, this work highlights new perspectives for hyperscanning research especially important considering growing concerns about current measures largely focused on interbrain synchrony.^55^^,^^56^^,^^57^^,^^58^ One major limitation of interbrain synchrony is that it can be driven by synchronized motor responses and artifacts between individuals performing together, a significant issue in the current study due to participants’ continuous eye and forearm movement tracking contaminating EEG. Such contamination inflates interbrain coherence between the two participants, reflecting the degree of movement synchrony between the two participants rather than neural synchrony.^55^^,^^57^ Importantly, consistent with the argument that understanding the neural bases of joint action requires addressing questions at different levels of analysis,^59^ our results emphasize the complementarity of approaches focused on individual brains to better understand the neural processes supporting social behaviors.

### Limitations of the study

While our findings provide insights into the neural tracking of self-other error, several limitations should be acknowledged. Firstly, the observed coherence measures are correlational in nature, precluding definitive conclusions about causal relationships related to top-down and bottom-up processes along the dorsal visual stream. The coherence and time lag analyses used in this study are limited in their ability to distinguish predictive coding from alternative alignment processes, warranting caution in interpreting the results. The leadership effects we report could potentially reflect other processes, such as differences in sensory sampling or strategy, rather than distinct neural top-down and bottom-up processes. Secondly, although our control analyses suggest minimal contribution of participants’ forearm and eye movements (see supplemental information), we cannot fully exclude the possibility that these movements contributed to some of the observed effects. Lastly, while our results are consistent with predictive processing frameworks, they do not provide direct evidence for causal mechanisms. Future studies employing neuroimaging techniques enabling robust source estimation and effective connectivity analyses or perturbation protocols could help gain more direct evidence of predictive processing. Specifically, consistent with other studies on interpersonal coordination,^22^^,^^25^^,^^26^^,^^60^ future research could incorporate brain stimulation to directly test the causal role of specific brain regions in self-other error tracking and underlying leader-based predictive processes.

To conclude, the present study provides new insight into the neural processes underlying visuo-motor interpersonal synchronization. We show in experimental settings where interpersonal synchronization is not pre-planned but improvised that the human brain continuously tracks and processes visual self-other synchronization error through dynamic selective neural processes modulated by an individual’s leadership role. Our results suggest a central role of the dorsal visual stream in the processing of self-other error, which might contribute to balancing predictive and adaptive processes, possibly doing so more optimally when there is no designated leader. Alignment in brain dynamics and self-other processing across individuals is the key for effective interpersonal synchronization and successful social interaction through visual information.

## Resource availability

### Lead contact

Requests for further information and resources should be directed to and will be fulfilled by the lead contact, Manuel Varlet (m.varlet@westernsydney.edu.au).

### Materials availability

This study did not generate new unique reagents.

### Data and code availability

Data and code are available on the open science framework at https://osf.io/7zvna/. Further information is available in the key resources table and will be provided by the lead contact on request.

## Acknowledgments

This study was supported by the 10.13039/501100000923Australian Research Council (DP170104322, DP220103047).

## Author contributions

M.V.: conceptualization, methodology, data analysis and interpretation, and writing—original draft, S.N.: conceptualization, data interpretation, and writing – review and editing, P.E.K.: conceptualization, data interpretation, and writing – review and editing.

## Declaration of interests

The authors declare no competing interests.

## STAR★Methods

### Key resources table

**Table:** 

REAGENT or RESOURCE	SOURCE	IDENTIFIER
**Deposited data**		

Preprocessed EEG Data	This paper	OSF: https://osf.io/7zvna/

**Software and algorithms**		

Matlab R2023a	MathWorks	https://au.mathworks.com/products/matlab.html
FieldTrip	Oostenveld et al.^37^	https://www.fieldtriptoolbox.org
Analysis Scripts	This paper	OSF: https://osf.io/7zvna/

### Experimental model and study participant details

#### Participants

The participants were 56 individuals (36 females and 20 males), aged between 18 and 45 years old (M = 24.70; SD = 5.63), tested by.^29^ These individuals had been randomly matched to form 28 pairs of participants, 11 female-female, 3 male-male, and 14 female-male pairs in total. All participants had normal or corrected-to-normal vision, were right-handed, and provided written informed consent prior to the experiment. The study received approval from the Human Research Ethics Committee at Western Sydney University.

### Method details

#### Procedure

After providing their consent, participants were seated in two chairs in front of each other and fitted with EEG electrodes and motion capture sensors on their right index fingers to record their brain activity and movement while performing the joint synchronisation task. Participants were instructed to perform continuous forearm oscillations together that were synchronised (mirrored each other) along the horizontal axis, and importantly, that were novel by continuously adjusting movement speed and amplitude,^28^ as illustrated in Figure 1. This task necessarily entails continually watching each other’s movements to maintain interpersonal synchronisation. The chairs were positioned to ensure that participants moved comfortably while their index fingers were kept extended as close as possible without contact. Participants’ movements were monitored by the experimenter to ensure that instructions were followed and that novel motion patterns were produced throughout the experiment.

The task was performed by each pair of participants in three different conditions – the Follower-Leader and Leader-Follower conditions, in which Leader and Follower roles were assigned to participants, and the Joint condition, in which no leadership roles were assigned. After one practice trial in each of the three conditions, the participants performed 12 trials of 180 s (i.e., 4 trials in each condition) presented in a randomised order. The experiment lasted approximately 100 min, including EEG preparation and breaks. See^29^ for further details, including flickering LEDs attached to participants’ index fingers to tag self and other movement information at frequencies above those of interest in the current study.

### Quantification and statistical analyses

#### Self-other error synchrony

A Polhemus LIBERTY motion tracker (Polhemus Ltd., VT, USA) was used to record the index finger movements of the two participants with 0.01 mm spatial resolution while performing the synchronisation task. Movement data were upsampled from the 240 Hz original sampling rate to 500 Hz to enable analyses with EEG data. Self-other synchronisation error was then calculated as the absolute position difference between the two participants’ movement along the main axis of variance extracted using a Principal Component Analysis (PCA) to control for deviations from the motion tracker’s horizontal axis (see Figure 1).

#### EEG recording and analyses

A Biosemi Active-Two system (Biosemi, Amsterdam, Netherlands) with 64 Ag-AgCl electrodes placed over the scalp of the two participants of the pairs according to the international 10/20 system was used to record brain activity at a sampling rate of 2048 Hz.

##### Preprocessing

Data were high-pass filtered at 0.1 Hz, notch filtered to remove 50 Hz (and corresponding harmonics) electrical power contamination, and downsampled to 500 Hz. Faulty channels were interpolated with the neighbouring channels (i.e., an average of 1.7 [*SD* = 1.41] interpolated electrodes per participant and never more than 5 electrodes). Eye blink artefacts were removed using an Independent Component Analysis (FastICA), as implemented in Fieldtrip.^37^ One single component related to eye blinks was removed per participant (no component identified and removed for 8 participants) based on visual inspection of the topography and time-course. No attempt to remove eye-tracking artefacts (i.e., synchronised eye movements with self- and other-generated movements) was made, as they had no critical bearing on the analysis of the neural tracking of self-other error (see supplemental information for control analyses). EEG data were then re-referenced to the average of all scalp electrodes.

##### EEG-error coherence

EEG-Error coherence was used to capture the alignment of brain activity with continuous fluctuations in self-other synchrony error across different frequencies.^30^^,^^31^ Cross-spectral coherence analyses have been extensively used in previous research to successfully capture phase alignment between neural signals,^61^^,^^62^ between movement signals,^63^^,^^64^ and between neural and movement signals.^65^^,^^66^^,^^67^ Coherence is essentially the frequency-domain counterpart of cross-correlation in the time domain. It quantifies how much one signal can explain the variance in another signal at different frequencies, similar to how a correlation coefficient works in the time domain.^61^^,^^68^ While correlation analyses can be dominated by low-frequency components with large amplitudes, coherence analyses can help normalise signal power across frequencies, enabling a more detailed examination of signal relationships at specific frequency bands. Cross-spectral coherence was computed between all EEG channels and self-other error. Fast Fourier Transform (FFT) with a Hanning window was used on 5 s epochs with 50% overlap to obtain power and phase estimations for each EEG channel and self-other error from 0 to 10 Hz with a frequency resolution of 0.2 Hz. The 0-10 Hz range and 5 s epochs enabled sufficient frequency resolution to examine lower frequency modulations, typical of human movement synchronisation,^69^ and 50% overlap or more is often standard as it enables to significantly improve coherence estimations.^70^ We used a multitaper approach to improve coherence estimation,^31^^,^^71^^,^^72^ as implemented in Fieldtrip. Three tapers were used, resulting in spectral smoothing of ±0.3 Hz. Coherence was computed as:

$${Coh}_{EEG-Error}\left(f\right)=\frac{\left|S_{EEG-Error}\left(f\right)\right|}{\sqrt{S_{EEG}\left(f\right)S_{Error}\left(f\right)}}$$

where S_EEG_ (f) and S_Error_ (f) correspond to the autospectral density of the EEG channel and self-other error, and S_EEG-Error_(f) corresponds to the cross-spectral density.^61^^,^^68^ This resulted in one coherence value between 0 and 1 for each frequency from 0 to 10 Hz, with 0 indicating no synchrony between EEG and self-other error and 1 indicating perfect synchrony.

Coherence was also computed on permuted data by shuffling self-other error timeseries across trials within conditions to obtain a baseline level of coherence values.^30^^,^^31^ We performed 9 permutations in total corresponding to all possible combinations of the 4 trials within each condition, and then averaged all permutations and conditions to obtain a single baseline value for each frequency and participant. This baseline was used to differentiate genuine alignment between EEG and self-other times series from alignment occurring by chance and trivially driven by the error power spectrum.^30^^,^^55^^,^^61^^,^^72^

##### Source space analyses

Cross-spectral coherence was also computed in source space to examine brain regions underlying the neural tracking of visual self-other error. Data in the source space was calculated in Fieldtrip using LCMV and a template standard head model (Montreal Neurological Institute; MNI) with a 1 cm grid.^37^ LCMV filters were computed for each voxel with 7% regularisation using the covariance matrix calculated over all trials of the three leadership roles. Coherence was then computed on these time series as in channel space except that coherence was calculated for 201 time lags of self-other error, from -2 s to 2 s with a 0.02 s step,^73^ to examine the time course of the neural processing of visual self-other error and its underlying predictive mechanisms. Coherence was averaged within the 0-6 Hz frequency range, which showed a significant difference between real and permuted data in channel space, to avoid computing overload.

Coherence data were also averaged within five regions of interest (ROIs) – V1, Cuneus, Precuneus, Paracentral lobule, and SMA – using the AAL (Automated Anatomical Labeling) atlas^38^ to examine the time course of EEG-Error coherence from early visual cortex to motor regions through the dorsal visual stream for the different leadership roles. We selected the AAL atlas for its balanced parcellation scheme, which provides sufficient regional detail while accounting for the limited spatial resolution inherent to EEG. We extracted for each ROI and participant the maximum coherence value and its corresponding time lag to examine differences in coherence magnitude and timing across leadership roles. To control for potential contributions of motor activity related to movement execution, we also calculated the coherence for the different time lags between changes in participants’ instantaneous movement frequencies and self-other error. As with self-other error, instantaneous movement frequency remains constant if there is no change in forearm oscillations (contrary to movement position and its derivatives, and eye movement contaminating EEG), and therefore, best captures changes in movement alignment with self-other error that might contribute to changes in EEG-Error coherence. Participants’ instantaneous frequencies were calculated as the temporal rate of change of continuous phase computed using the Hilbert Transform, and we then took the absolute values of the derivative to capture changes irrespective of their direction (i.e., acceleration or deceleration). Topographies and source distributions presented in the figures were made using Letswave6^74^ and BrainNet.^75^

#### Statistical analyses

All statistical analyses were performed with R version 4.3.3. Repeated-measures ANOVAs were conducted with the package “afex” version 1.3-1 with Greenhouse-Geisser correction applied when the assumption of sphericity was violated.^76^ Pairwise contrasts were used to examine the significant effects further, with Bonferroni adjustment for multiple comparisons. We also calculated Bayes factors to quantify the evidence in favour of the alternative hypothesis over the null hypothesis (BF_10_), as implemented in the package BayesFactor for R.^77^ Throughout the results our interpretation was informed by converging evidence from both frequentist and Bayesian statistical approaches, allowing for a comprehensive understanding of the data. Significant *p* values (< 0.05), and BF_10_ > 10, providing strong evidence in favour of the alternative hypothesis, were considered converging evidence. Graphics were made with the package ggplot2.^78^^,^^79^

Cluster-based permutation testing^37^ was also used to test significant differences in the channel space along the different frequencies. We ran point-by-point paired *t*-tests to examine (i) differences in coherence between real (averaged across the three leadership roles) and permuted data, and (ii) differences in power between designated and undesignated leadership roles. We also ran point-by-point correlation to test (iii) relationships between EEG-Error coherence and the magnitude of visual self-other error. We then determined clusters of adjacent frequencies above the critical *t*-value for a parametric two-sided test and the magnitude of each cluster by calculating the sum of the absolute *t*-values constituting each cluster. We used 1000 random permutations (random sign changes) to obtain a reference distribution of maximum cluster magnitude. The proportion of random partitions that resulted in a larger cluster-level statistic than the observed one (*p* value) was calculated. Clusters in observed data were considered as significant if their magnitude exceeded the threshold of the 95^th^ percentile of the permutation distribution.
