# Supplementary material for: Dynamic neural processing of self-other synchronization error in interpersonal coordination
Source: iScience. 2025 Nov 17;28(12):114081. doi: 10.1016/j.isci.2025.114081 (PMC12719764; doi:10.1016/j.isci.2025.114081)
Supplement: Document S1. Figures S1–S4 [file mmc1.pdf]

iScience, Volume *28*

Supplemental information

Dynamic neural processing of self-other synchronization error in interpersonal coordination

Manuel Varlet, Sylvie Nozaradan, and Peter E. Keller

Eye-tracking and its influence on EEG data and coherence analyses

This supplementary material provides details of participants' eye-tracking movements during the joint synchronisation task and of its influence on EEG data and coherence analyses. The figures presented below show that participants' eye movements were highly synchronised with self- and other-generated movements, making it challenging, if not impossible, to examine their neural processing without using frequency-tagging techniques, as employed in Varlet et al. (2020)'s original study.

High coherence with self- and other-generated movements is not only observed for Electrooculography (EOG) but also Electroencephalography (EEG). High synchrony between eye movement and self- and other-generated movements is evident in EEG across the scalp, with the strongest coherence observed in fronto-lateral channels around the eyes. In contrast, self-other error is characterised by coherence of lower magnitude distributed over occipital and parietal regions, compatible with neural activity from visual and motor regions, as detailed in the manuscript. In addition, the figures below show that focusing on the changes in the instantaneous frequency of participants' movements rather than their position enables significant decoupling from eye movements and emphasising motor- and visual-related neural activity in EEG of much lower magnitude.

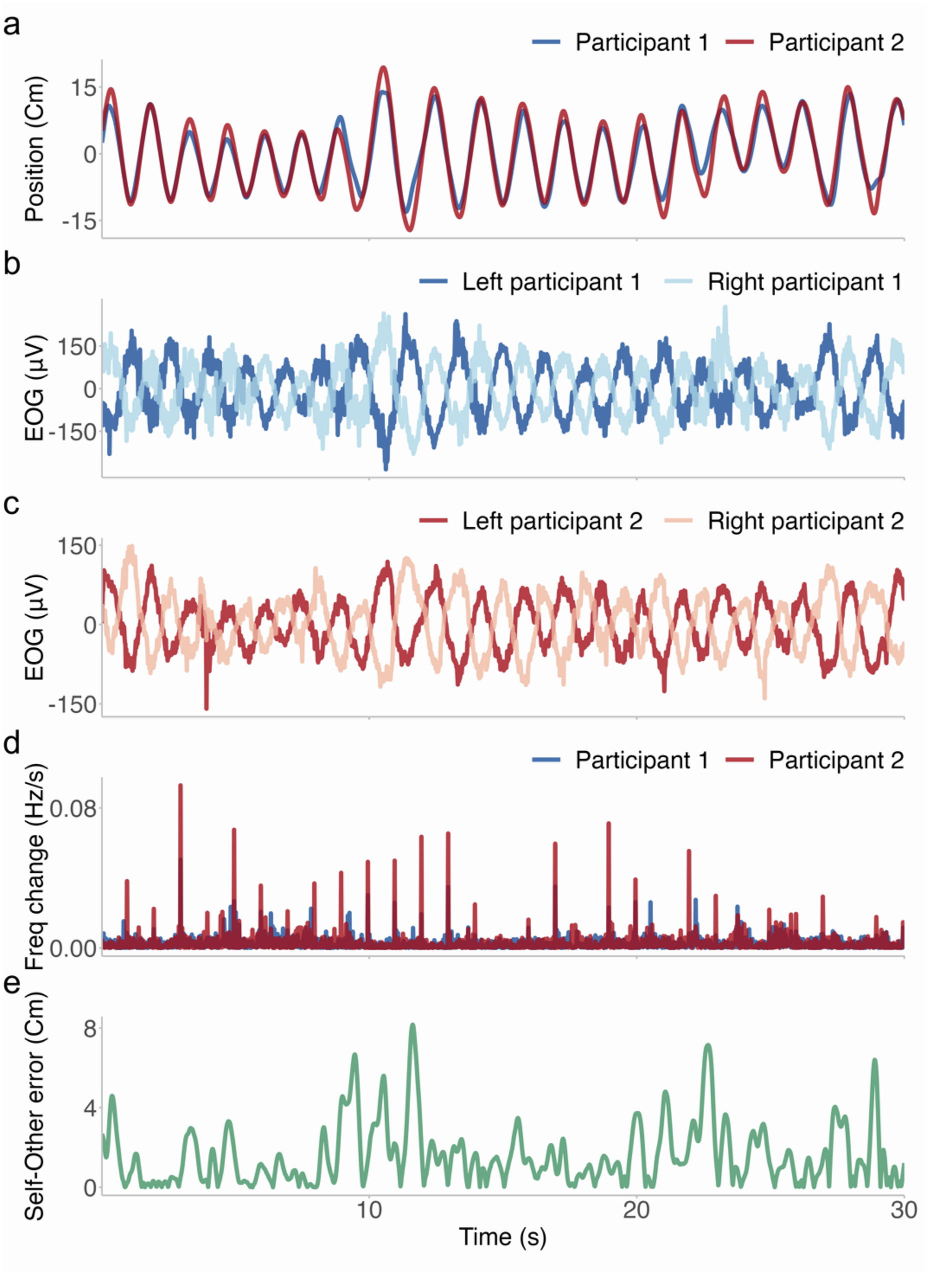

Figure 1. 30-s representative time series of two participants' movements (blue and red in a) and the corresponding self-other error (green in e) presented in the manuscript (Figure 1), with the addition of participants' corresponding Electrooculography EOG (left and right sides, panels b and c) and changes in their instantaneous movement frequency (blue and red in d).

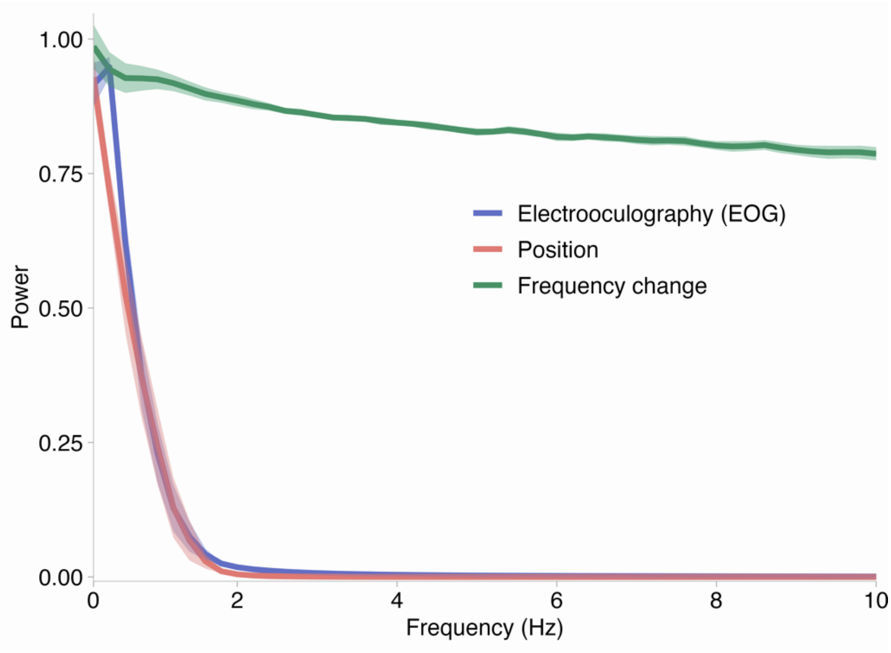

Figure 2. Power spectrum of Electrooculographic data (EOG, averaged from both eyes), participants' position and changes in instantaneous movement frequency. Power spectra are calculated from 0 to 10 Hz with a 0.2 Hz resolution (see Methods for details) and normalised between 0 and 1 to enable comparison between the different types of datasets. The results show a similar power distribution between EOG and position data, with large low-frequency magnitude and reduced amplitude above 2 Hz, whereas the power for frequency change data is more distributed across the different frequencies. Coloured shaded areas represent 1 × 95% CI of the mean computed for within-subject designs (Morey, 2008).

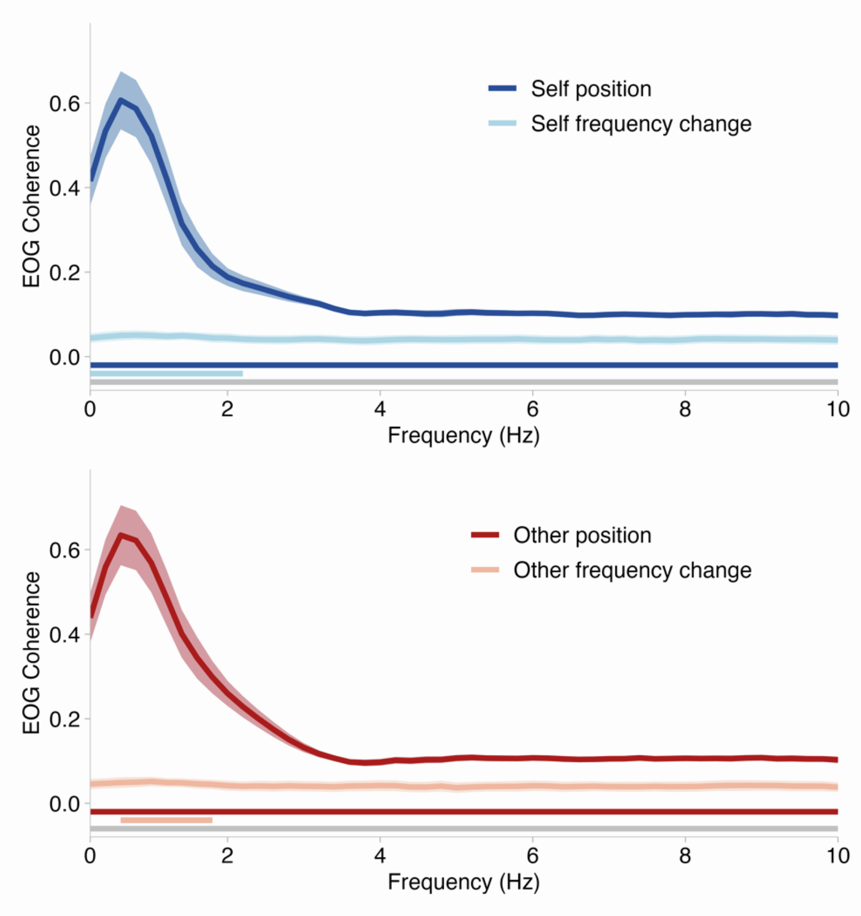

Figure 3. Coherence between EOG and the position and changes in instantaneous frequency of participants' self-generated movement (top panel) and of partners' (other-generated) movement (bottom panel). Coherence (averaged from both eyes) is calculated from 0 to 10 Hz with a 0.2 Hz resolution (see Methods for details). Coloured shaded areas represent 1 × 95% CI of the mean computed for within-subject designs (Morey, 2008). Blue and red horizontal lines indicate clusters of coherence values significantly higher than permuted data. Grey horizontal lines indicate clusters of coherence values significantly higher for position than frequency change data. The results reveal strong synchronisation between EOG and position data for both self- and other-generated movements. Coherence between EOG and frequency change data is significantly lower across all frequencies compared to position data, with low magnitude overall and no values significantly higher than permuted data beyond 2.2 Hz.

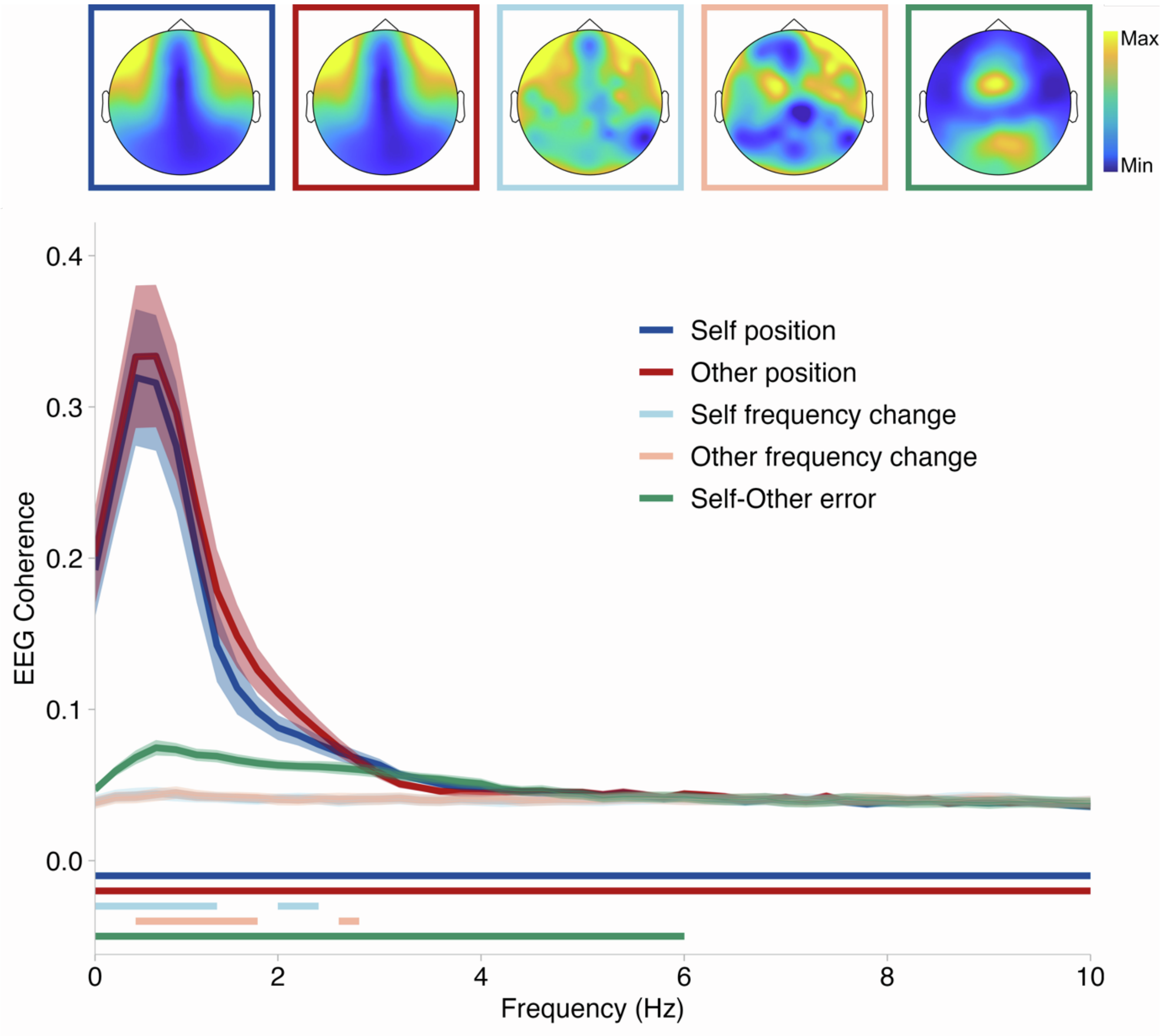

Figure 4. Coherence between EEG and the position and changes in instantaneous frequency of participants' self-generated and partners' (other-generated) movement, and self-other error. Coherence averaged across all electrodes is depicted from 0 to 10 Hz with a 0.2 Hz frequency resolution (see Methods for details), with coloured shaded areas representing 1 × 95% CI of the mean computed for within-subject designs (Morey, 2008). Coloured horizontal lines indicate clusters of coherence values significantly higher than permuted data. Topographical maps depict coherence averaged within 0-6 Hz, corresponding to the significant frequency range for self-other error. The results show high synchrony between EEG and position data, due to participants' eye tracking movements, evident in maximum coherence distributed around the eyes. Coherence with frequency change data is much weaker, showing a reduced influence of eye movements and emphasising motor and visual-related neural activity from frontal, occipital and parietal regions. Coherence with self-other error and its topographical distribution contrasts with position and frequency change data, further supporting the involvement of distinct neural processes.
